# The effects of smoking on pain scores, vital signs, and analgesic consumption in patients undergoing tympanomastoidectomy surgery

**DOI:** 10.18332/tid/189301

**Published:** 2024-06-17

**Authors:** Murat Tekin, Kadriye B. Ceylan, Murat Ozturk

**Affiliations:** 1 Department of Anesthesiology and Reanimation, Kocaeli University, Kocaeli, Türkiye; 2 Department of Otorhinolaryngology, Kocaeli University, Kocaeli, Türkiye

**Keywords:** smoking, pain scales, postoperative pain, tympanomastoidectomy, pain control

## Abstract

**INTRODUCTION:**

In this study, we investigate the effects of smoking on pain scores, vital signs, and analgesic consumption in the intraoperative and postoperative period in patients undergoing tympanomastoidectomy surgery.

**METHODS:**

A total of 100 patients with American Society of Anesthesiologists I-II status, aged 18–55 years, and who were planned to undergo tympanomastoidectomy surgery were divided into two groups: smokers (Group 1) and non-smokers (Group 2). The patients were compared for preoperative, intraoperative, and 24-hour postoperative carboxyhemoglobin, blood pressure, oxygen saturation, respiratory rate, heart rate, pain intensity and verbal numerical rating scales, the extent of patient-controlled tramadol dose, nausea, and vomiting.

**RESULTS:**

There were 50 individuals in each group. Postoperative analgesic consumption and pain scores were higher in Group 1, and the first postoperative pain was felt earlier. Furthermore, in Group 1, preoperative carboxyhemoglobin levels and postoperative nausea were statistically higher before, after, and at the tenth minute after induction, whereas oxygen saturation was lower. The two groups had no statistical difference regarding intraoperative and postoperative vital signs. Postoperative analgesic consumption was not affected by age or gender.

**CONCLUSIONS:**

Smoking changes postoperative pain management, especially for this kind of operation, and these patients feel more pain and need more postoperative analgesic doses. Therefore, effective postoperative pain control should take account of smoking behavior, and analgesic doses may need to be adjusted for patients who smoke.

## INTRODUCTION

Postoperative pain is an acute pain that begins with surgical trauma and gradually decreases with tissue healing. Moreover, there is a relationship between smoking and this pain. The effects of smoking on anesthesia and pain are complex and not well understood. However, it is known that cigarettes contain nicotine and that has analgesic properties, and this has been demonstrated in visceral pain models^1^. Nicotine may affect many physiological systems due to its pharmacological characteristics. It affects the peripheral and central nervous systems (CNS), cardiovascular and gastrointestinal systems, and exocrine glands by activating nicotine-specific receptors and releasing many different neuromediators. It has been shown that chronic nicotine usage increases the perception of pain, and acute nicotine use provides an analgesic effect^2,3^. While it may have an analgesic effect on the CNS at low doses, it may cause addiction-related tremors and seizures in high doses.

With sudden smoking cessation, hyperalgesia may occur after surgery or painful stimulation, and the pain threshold is decreased. In this study, observationally, we aimed to compare postoperative analgesic consumption in adult smokers and non-smokers who were undergoing tympanomastoidectomy surgery and did not have any other systemic problems. Patients who would undergo tympanomastoidectomy were chosen because it is a relatively stable and standardized procedure, not related to the airway, and has similar incision and surgical content. These patients are routinely evaluated for any upper respiratory tract pathologies that may interfere with the anesthesia procedure or postoperative pain.

## METHODS

### Study design and participants

Adult patients aged 18–55 years and with an American Society of Anesthesiologists (ASA)^4^ anesthetics risk grading of I-II and who would undergo standard tympanomastoidectomy surgery and volunteered to participate were included in the study. Any accompanying systemic disease was ruled out by preoperative detailed physical examination, chest scan, electrocardiography, routine blood tests, and patient’s history and records. The study aimed to observe whether there was a difference between the 24-hour postoperative analgesic requirements between smokers and non-smokers and, if there was a difference, what percentage difference there was. Exclusion criteria were patients: with chronic pain, who were unable to cooperate, who smoked during hospitalization in the postoperative period, who smoked 24 hours postoperatively, with an anesthesia risk of ASA III and above, and who did not want to participate in the study.

The patients were divided into: smokers (Group 1) and non-smokers (Group 2). Group 1 consisted of patients who smoked at least ten cigarettes a day, smoked for at least one year, and continued to smoke until one week before the operation. Group 2 consisted of patients who had never smoked before or had smoked but had not smoked for at least six months. The sample size calculated with α=0.01 error and 95% working power was determined as 48 patients for each group, and it was planned to include 50 patients for both groups in the study, thus a total of 100 patients.

### Variables and procedures

A detailed smoking history was taken when the patients were admitted to the Ear, Nose, and Throat service. The smoker group was asked not to smoke during hospitalization and was informed about the study. All patients and their accompanying relatives were informed how to use the Patient Controlled Analgesia (PCA)^5^ device after the surgery. They were also informed about the Verbal Numerical Rating Scale (VNRS)^6^, which ranges from 0 = ‘no pain’ to 10 = ‘unbearable pain’, and that it would be used to evaluate their pain in the postoperative period. Before the patients were taken to the operating room, peripheral vascular access was established with 20G intravenous cannulation, and premedication was administered with 0.03 mg/kg iv midazolam. After being taken to the operating room, DII lead Electrocardiography (ECG), heart rate (HR), non-invasive systolic blood pressure (SBP), diastolic blood pressure (DBP), mean blood pressure (MBP) and peripheral oxygen saturation (SpO_2_) were monitored. Initial values were recorded. The patients’ gender, age, height, weight, body mass index (BMI, kg/m^2^), additional diseases and medications used were noted. Carboxyhemoglobin (COHb) level was measured in venous blood before induction. Anesthesia induction was provided to both groups with 5–7 mg/kg thiopental, 1 μg/kg fentanyl, and 0.2 mg/kg mivacurium, and then they were intubated orotracheally, tidal volume 6–8 mL/ kg, frequency 10–12/min in controlled mode was ventilated to keep end-tidal carbon dioxide (CO_2_) values at 28–32 mmHg. Gas consisted of 40% O_2_, 60% air, 2% sevoflurane inhalation, and 0.1–0.2 μg/kg/min remifentanil infusion was used to maintain anesthesia.

Peripheral oxygen saturation, HR, SBP, DBP, and MBP were measured before and after induction at the 10th, 20th, and 30th minute and the 1st, 2nd, 3rd, and 4th hour. When subcutaneous stitching was started, 1 mg/kg tramadol and 0.1 mg/kg ondansetron were administered. Postoperative tramadol PCA was prepared. PCA consisted of 5mg/mL tramadol in 100 mL of 0.9% NaCl. The bolus dose was 10 mg, lockout time was 10 minutes, and maximum pressure for one hour was set at 6 (?).

After the patients woke up and were taken to the recovery room, the use of PCA was explained again. Patient-reported VNRS, tramadol consumption, nausea, vomiting, and vital signs were monitored at the 1st minute of postoperative pain and the 1st, 2nd, 6th, 12th, and the 24th hour, postoperatively.

The study was approved by the local ethics committee. Written informed consent was obtained from all patients. This study was performed in line with the principles of the Declaration of Helsinki.

### Statistical analysis

All statistical analyses were performed using IBM SPSS for Windows version 20.0 (IBM Corp., Armonk, NY, USA). Kolmogorov-Smirnov’s test was used to assess the assumption of normality. Since the normality assumption held, continuous variables are presented as mean ± standard deviation (SD). Categorical variables were summarized as counts and percentages. Comparisons between groups were carried out using independent samples t-test. Pearson’s correlation analysis determined associations between continuous variables. The chi-squared test examined associations between categorical variables. All hypothesis tests were conducted as two-sided. A p<0.05 was considered statistically significant.

## RESULTS

As planned, one hundred patients (51 female, 49 male), 50 in Group 1 and 50 in Group 2, were included in the study. The patients’ ages ranged 18–55 years, with a mean age of 35.19 years. The distribution of patients in terms of age, height, weight, BMI, and gender for Group 1 and Group 2 is presented in Table 1. There was no difference between the groups in terms of these demographic data.

**Table 1:** Comparison of the demographic data of the patients with American Society of Anesthesiologists I-II status, aged 18–55 years, who would undergo tympanomastoidectomy surgery, by smoking status (N=100)

	*Smokers (Group 1) Mean ± SD*	*Non-smokers (Group 2) Mean ± SD*	*p*
Age (years)	38.28 ± 12.06	32.10 ± 11.88	0.11
Height (cm)	169.43 ± 9.16	167.15 ±7.94	0.18
Weight (kg)	73.16 ± 17.63	67.62 ± 12.87	0.07
Body mass index (kg/m^2^)	25.17 ± 4.66	24.04 ± 3.67	0.99
Gender (Male/Female)	27/23	22/28	0.24

*Statistical tests: chi-squared for age and gender; independent sample t-test for the others.

### Total surgical times and basal COHb values

The mean ± standard deviation (SD) total surgery time was 3.65 ± 0.58 hours for Group 1 and 3.65 ± 0.66 hours for Group 2 (p=0.99). The mean basal COHb value was 1.98 ± 0.08% for Group 1 and 0.70 ± 0.03% for Group 2 (p<0.01).

### Peripheral oxygen saturation, heart rate, and blood pressure measurements

When intraoperative SpO_2_ was compared for Group 1 and Group 2, the Group 1 pre-induction, post-induction, and tenth-minute SpO_2_ values were significantly lower than in Group 2 (Table 2). After this time point, no significant differences were found. In terms of HR and MBP, no difference was found at any time point between the groups. Furthermore, no differences were detected between the groups for either SBP or DBP.

**Table 2:** Perioperative, intraoperative, and postoperative peripheral mean oxygen saturation, heart rate, and blood pressure, for patients with American Society of Anesthesiologists I-II status, aged 18–55 years, who would undergo tympanomastoidectomy surgery, divided into smokers (G1) and non-smokers (G2) (N=100)

	*Peripheral oxygen saturation (%)*			*Heart rate (beats/min)*			*Mean blood pressure (mmHg)*		
	*G1*	*G2*	*p*	*G1*	*G2*	*p*	*G1*	*G2*	*p*
Pre induction	98.20 ±0.24	99.3 ± 0.15	<0.01	81.55 ± 14.45	82.26 ± 13.07	0.79	96.77 ± 12.95	96.50 ± 15.46	0.89
Post induction	99.44 ± 0.27	99.71 ± 0.98	0.02	80.87 ± 14.43	83.03 ± 15.80	0.47	89.44 ± 16.35	87.50 ± 18.58	0.57
10th minute	98.22 ±0.51	99.52 ± 0.20	0.01	72.79 ± 12.56	77.05 ± 13.59	0.10	80.12 ± 14.83	81.09 ± 11.87	0.71
20th minute	98.44 ± 0.51	99.4 ± 0.18	0.12	68.65 ± 11.08	71.33 ± 12.03	0.24	74.30 ± 13.72	75.15 ± 12.12	0.74
30th minute	98.57 ± 0.50	99.38 ± 0.21	0.06	65.16 ± 8.99	68.20 ± 10.46	0.11	72.77 ± 11.92	72.36 ± 11.73	0.87
1st hour	98.50 ± 0.49	99.38 ± 0.25	0.11	62.28 ± 7.08	65.91 ± 9.62	0.07	69.38 ± 10.15	71.92 ± 10.94	0.21
2nd hour	99.11 ± 0.33	99.71 ± 0.15	0.09	67.00 ± 8.49	67.15 ± 10.63	0.93	74.85 ± 8.77	76.77 ± 11.88	0.36
3rd hour	99.27 ± 0.23	99.80 ± 0.14	0.09	67.55 ± 8.28	68.90 ± 11.80	0.50	72.97 ± 8.57	77.13 ± 13.56	0.07
4th hour	99.33 ± 0.25	99.80 ± 0.14	0.07	67.55 ± 10.78	67.71 ± 8.98	0.96	75.27 ± 10.70	74.00 ± 10.56	0.71

*Independent sample t-test was used for the statistical analysis.

### Postoperative verbal numerical rating scale ( VNRS )

**Figure 1:**
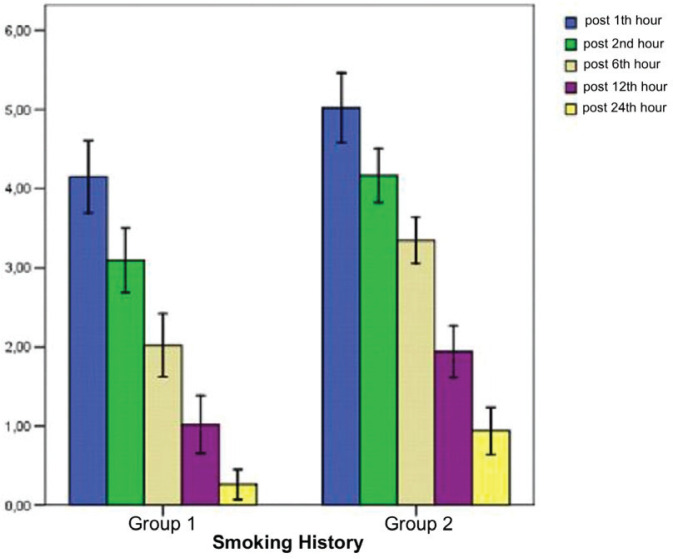
Postoperative mean verbal numerical rating scales of patients with American Society of Anesthesiologists I-II status, aged 18–55 years, who would undergo tympanomastoidectomy surgery, divided into smokers (Group 1) and non-smokers (Group 2) (N=100)

When the mean postoperative VNRS values were compared between Group 1 and Group 2, the values at all time points assessed were significantly higher in Group 1 compared to Group 2 (Table 3 and Figure 1).

**Table 3:** Postoperative mean verbal numerical rating scale values and mean postoperative tramadol consumption in patients with American Society of Anesthesiologists I-II status, aged 18–55 years, who would undergo tympanomastoidectomy surgery, divided into smokers (G1) and non-smokers (G2) (N=100)

	*Postoperative verbal numerical rating scale score*			*Postoperative tramadol consumption (mg)*		
	*G1*	*G2*	*p*	*G1*	*G2*	*p*
1st hour	5.11 ± 0.36	4.28 ± 0.40	<0.01	18.16 ± 8.58	14.07 ± 8.96	0.02
2nd hour	4.27 ± 0.17	2.95 ± 0.39	<0.01	75.51 ± 36.34	43.77 ± 23.47	<0.01
6th hour	2.94 ± 0.18	1.95 ± 0.37	<0.01	168.36 ± 71.10	76.75 ± 34.47	<0.01
12th hour	2.05 ± 0.22	1.19 ± 0.28	<0.01	278.77 ± 103.07	115.62 ± 55.98	<0.01
24th hour	1.11 ± 0.25	0.57 ± 0.20	<0.01	353.67 ± 108.06	200.11 ± 48.55	<0.01

*Independent sample t-test was used for the statistical analysis.

### Postoperative tramadol consumptions

**Figure 2:**
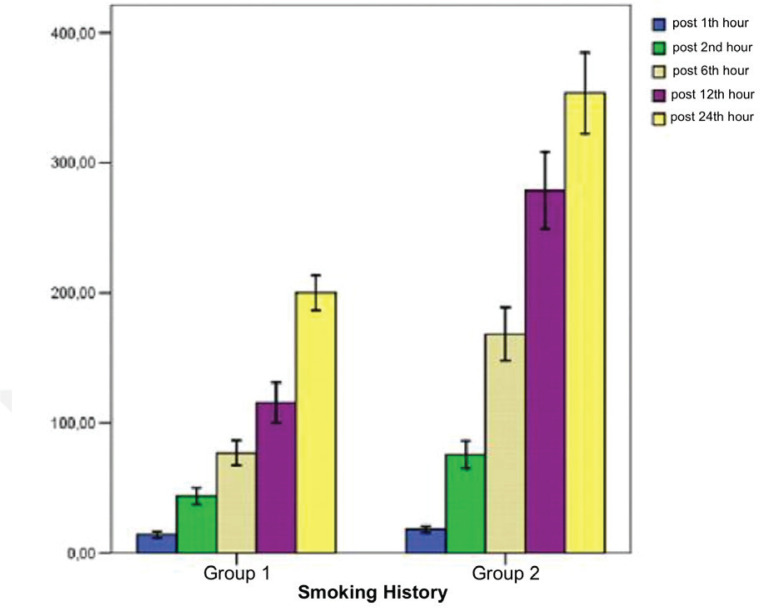
Postoperative tramadol consumptions of patients with American Society of Anesthesiologists I-II status, aged 18–55 years, who would undergo tympanomastoidectomy surgery, divided into smokers (Group 1) and non-smokers (Group 2) (N=100)

When the postoperative tramadol consumptions between Group 1 and Group 2 were compared, the consumption in Group 1 was significantly greater than in Group 2 at all time-points (Table 3 and Figure 2). When tramadol consumption by age was compared, no correlation was found (r= -0.009, p=0.51) between age and postoperative 24-hour total tramadol consumption in Group 1. Similarly, when tramadol consumption by gender was assessed in Group 1, the distribution of postoperative 24-hour total tramadol consumption was similar between women (348.72 ± 109.54) and men (334.07 ± 104.78). Statistically, there was no significant difference between them (p=0.17).

### Postoperative time to the first report of pain

When the time to feeling the first postoperative pain was compared between Group 1 and Group 2, the mean was 13.83 ± 11.52 minutes in Group 1 and 30.67 ± 16.30 minutes in Group 2 (p<0.01).

### Postoperative respiratory rate and heart rate

When postoperative respiratory rates were compared between Group 1 and Group 2, there were no differences between the rates for the first, second, sixth, twelfth, and twenty-fourth hours (p=0.68, p=0.48, p=0.18, p=0.28, and p=0.23, respectively). Similarly, when postoperative HRs were compared between the groups at the same time points, no difference was found at any time point measured ( p=0.20, p=0.40, p=0.26, p=0.19, and p =0.96).

### Postoperative systolic, diastolic, and mean blood pressure

When postoperative SBP, DBP, and MBP were compared between Group 1 and Group 2, no significant differences were found at the first, second, sixth, twelfth, or twenty-fourth hours (SBP: p=0.43, p=0.65, p=0.09, p=0.89, and p=0.92; DBP: p=0.26, p=0.07, p=0.35, p=0.37, and p=0.58; MBP: p=0.31, p=0.14, p=0.21, p=0.73, and p=0.77).

### Postoperative nausea and vomiting

Nausea was observed in two (4.0%) patients in Group 1 and 8 (16.0%) patients in Group 2 (p=0.02). Vomiting was observed in 2 (4.0%) patients in Group 1 and 3 (6.0%) patients in group 2 (p=0.18).

## DISCUSSION

Smoking is an important parameter for surgery, anesthesia, and pain management of patients. Therefore, smoking status should be questioned carefully in patients undergoing elective surgery and associated anesthetic procedures. Patients who were undergoing elective tympanomastoidectomy were selected for several reasons, including a relatively standard incision and operative procedure, being relatively separated from factors that may affect pain, the frequent examination of the surgical area and airway in the perioperative period, and the higher rate of detection of possible influencing factors. It was thought that this would increase the reliability of the study’s results. Kay-Rivest et al.^7^ studied the association between smoking and 30-day outcomes in otologic surgery, especially for adverse events but not pain.

It has been shown that nicotine, the most important active substance in cigarette smoke and the agent responsible for the effects of smoking, has analgesic effects. It has been reported that smoking even one cigarette reduces awareness and increases tolerance to some experimental painful stimuli^8^. Moreover, the analgesic effect of cigarettes is not seen in cigarettes depleted of nicotine^9^. Nicotine exerts its analgesic effect through nicotinic acetylcholine receptors (nAChRs), and supraspinal and spinal activation of nAChRs results in opioid release^10^.

While there are studies suggesting that smoking should be stopped at least eight weeks before operations to minimize postoperative complications^11,12^, Warner et al.^13^ reported that smokers who quit smoking six months before an operation would be similar to non-smokers in terms of postoperative complications. Zhao et al.^14^ found that preoperative smoking cessation at least three weeks before surgery led to better postoperative pain outcomes, whereas Kamma et al.^15^ showed that patients who quit smoking at least one month preoperatively had better pain scores than current smokers. Based on these findings, in the present study, those who had not smoked for six months prior to the tympanomastoidectomy were included in the group consisting of non-smokers (Group 2). Furthermore, during the postoperative period, all patients stopped smoking for 24 hours.

In our study, tramadol PCA was used in the postoperative period, considering the mild-moderate VNRS values after the tympanomastoidectomy operation, and postoperative 24-hour tramadol consumption was 76.7% higher in smokers than in non-smokers. Compared to studies other than the narrow-scale study conducted by Marco et al.^16^, in our study, analgesic consumption was higher in smokers as a percentage. This may be due to differences in study population ethnicity, the use of tramadol versus morphine in the postoperative period for analgesia, different operations, and different periods for postoperative analgesic consumption. The city where this study was conducted is large, with an ethnically diverse population, which could support a more general conclusion. It is not known why smokers who quit smoking in the postoperative period consume more postoperative analgesics. This may be due to several reasons. One of these is that withdrawal symptoms such as insomnia and anxiety, which occur due to cessation of nicotine, are partially reduced by the sedation effect of opioids taken. Another possible reason may be due to the pharmacological relationship between smoking and opiates, especially because cigarette smoking induces opiate metabolism. It is known that smoking induces the CYP1A2 isoenzyme, one of the liver enzymes^17^. However, tramadol was used in our study, which is metabolized by CYP2D6, not CYP1A2^18^. Another explanation may be related to the pain control of smokers. Exogenous opioids and endogenous β-endorphins may relieve pain through opiate receptors, and tolerance to opioids means a higher requirement for opioids for pain control.

However, other factors, such as age, gender, and surgical procedures, may also affect postoperative analgesic consumption. While it has been reported that postoperative pain and analgesic requirements are inversely proportional to age^19^, there are also studies showing that women are more pain-tolerant and thus have less analgesic requirement^20^. There are also studies that, in contrast, show more pain reported by women than men^21^. The present study detected no differences between the two groups regarding age, height, weight, BMI, and gender. However, although it has been reported that analgesic consumption decreases as age increases, no correlation was found in the present study. This may have been because the patient groups were no older than 55 years and were relatively young. Different studies have investigated whether nicotine given systemically to patients will contribute to postoperative analgesia. Although studies indicate postoperative nicotine administration may be beneficial^22^, in placebo-controlled experiments, no significant effect was found^23^. Based on these data, the patients in our study were not given nicotine during the perioperative period.

Although smoking is thought to reduce acute pain, there are also studies showing that it increases chronic pain, including fibromyalgia, low back pain, and other painful conditions^24^. This may be due to the emergence of systemic diseases due to smoking, increased drug use, changes in receptor and hormone levels, depression, and psychosocial factors, all leading to chronic pain. That is why we did not include patients with chronic pain in the study. No significant difference was observed when we examined vital signs, such as intraoperative and postoperative SBP, DBP, MBP, HR, and postoperative respiratory rate.

In the present study, baseline COHb values were 182% higher in smokers than in non-smokers, as expected^25^. The difference in COHb values between smokers and non-smokers is caused by the high concentration of carbon monoxide (CO) in cigarette smoke. CO binds to hemoglobin with a higher affinity than oxygen and enters the pulmonary circulation, causing tissue hypoxia. This also explains why Group 1 (smokers) in the present study had lower SpO_2_ values than non-smokers. In the present study, SpO_2_ values in smokers before and after induction and at the intraoperative tenth minute were significantly lower than those of non-smokers. Low SpO_2_ may also be caused by the effects of smoking on the respiratory system, which range from impairing tracheobronchial clearance, reduced elasticity in the lungs, predisposition to emphysema, and eventual chronic obstructive airway disease.

Interestingly, the incidence of postoperative nausea was higher in non-smokers than in smokers in the present study. This may be because smoking increases the metabolism of volatile agents by inducing CYP2E1 enzymes, and the substances contained in cigarettes may have antiemetic properties^26^.

### Limitations

The present study has some limitations, which should be noted. Patients who had never smoked and those who quit smoking more than six months before the elective procedure could have been evaluated in separate groups but were combined in the present study. The present study aimed to evaluate groups more objectively by choosing a more standard surgery in terms of incision method and expected pain instead of choosing surgeries with higher postoperative pain expectations. Residual confounding not addressed by regression models, non-causal inference, and limited generalizability to other types of surgeries are other study limitations. The study population was of diverse ethnicity, but it still may not cover all ethnicities. Finally, larger group sizes would have allowed for a better investigation into the relationship between age and self-reported pain scores.

## CONCLUSIONS

In the present study, when a standardized surgical and patient group was examined, it was found that patients who smoked experienced more postoperative pain and earlier than non-smokers, and these findings were statistically significant. Furthermore, smokers had lower oxygen saturation and high COHb levels and also needed more self-administered postoperative analgesia, and these were also statistically significant. This study investigated the effects of smoking on the postoperative pain status of patients who had undergone a more standardized surgery that influenced postoperative pain less or none. Patient smoking status should be taken into consideration in terms of postoperative pain management.

## Data Availability

Data sharing is not applicable to this article as no new data were created.
